# Preconditioning rats with three lipid emulsions prior to acute lung injury affects cytokine production and cell apoptosis in the lung and liver

**DOI:** 10.1186/s12944-019-1137-x

**Published:** 2020-02-05

**Authors:** Li-Mi Huang, Qingqing Hu, Xiaoxia Huang, Yan Qian, Xin-He Lai

**Affiliations:** Department of Pediatrics, The First affiliated hospital of Wenzhou Medical University, Wenzhou, Zhejiang province China

**Keywords:** Acute lung injury, Parenteral nutrition, Cytokine, Apoptosis, Liver function

## Abstract

**Background:**

Critically ill patients are at higher risk having acute lung injury (ALI) and more often in need of parenteral nutrition. We sought to study whether preconditioning with representative of lipid emulsions for one week could benefit rats from ALI.

**Methods:**

Using a lipopolysaccharide (LPS)-induced ALI rat model and techniques such as polymerase chain reaction (PCR), enzyme-linked immunosorbent assay (ELISA), and terminal deoxynucleotidyl transferase dUTP nick end labeling (TUNEL) staining.

**Results:**

PGE_2_ production in the serum was highest in the LPS group, followed with Intralipid group, and the PGE_2_ level of these two groups was significantly (*P* < 0.05) higher than the rest. Intralipid conditioning caused significantly less production of LTB_4_ than the LPS, Clinoleic, or Omegaven group. In contrast to Intralipid, rats pretreated with Clinoleic or Omegaven significantly decreased their production of inflammatory mediators (IL-1 β, IL-6 and TNF-α), had less apoptosis in the lung tissues, and Omegaven greatly improved liver function upon lipopolysaccharide (LPS) challenge.

**Conclusions:**

In an ALI setting, preconditioning with Omegaven or Clinoleic was better than Intralipid in decreasing the intensity of the cytokine storm and apoptosis caused by LPS challenge, and Omegaven in addition had the potential to improve liver function. The results from the present study set a basis for further investigation of the molecular mechanisms of ALI, including the up- and downstream pathways of proinflammatory factor production, in search of (small) molecules intervening with the pathogenesis of ALI in order to translate relevant research findings into clinical benefit for patients with ALI. The use of Omegaven or Clinoleic, particularly in patients with ALI, is still characterized by uncertainty due to a lack of relevant studies. Future investigations must specifically focus on the route of administration and mode of application (enteral vs. parenteral/bolus vs. continuous), determining an optimal dose of Omegaven or Clinoleic, and the defining the best timepoint(s) for administration. Critically ill patients are at higher risk having acute lung injury (ALI) and more often in need of parenteral nutrition. The effect of lipid emulsion via parenteral nutrition on liver function was first time evaluated in rats in an ALI setting. The comparison of three forms of lipid emulsion in a rat model of acute lung injury was first time studied. The fish oil-based lipid emulsion decrease in PGE 2 and increase in LTB 4 was first time reported.

## Background

With an annual incidence of 20–85 per 100,000 and an overall mortality of 30–50% in the USA [1, 2], acute lung injury (ALI) is a heterogeneous syndrome of diverse etiologies that is characterized by intense and uncontrolled pulmonary inflammation in the early stages of this disease [3, 4]. ALI may evolve from septic shock, infection, tissue damage and trauma, among others. Lipopolysaccharide (LPS; also known as endotoxin), a structural component of the outer membrane of Gram-negative bacteria, is a key virulence factor in bacterial sepsis and other infections. Due to its importance in ALI pathogenesis, administration of LPS intratracheally in vivo is widely accepted as one of the most common models of ALI with a clinical relevance [5, 6].

Critically ill patients, such as those with acute respiratory distress syndrome (the more severe form of ALI) often require parenteral nutrition, and lipid emulsions are crucial for providing essential fatty acids [7–10], but with the associated risk of causing parenteral nutrition-associated liver disease [11–13]. Among the lipid emulsion brands approved for parenteral nutrition, Intralipid and Omegaven are almost pure soybean or fish oil (FO) products, whereas Clinoleic contains 80% olive oil and 20% soybean oil [14].

Supplementation of fatty acids has long relied exclusively on soybean oil, which contains a large amount of linoleic acid (LA, 18:2), a n-6 polyunsaturated fatty acid (PUFA), which is a precursor of arachidonic acid (AA). Rapid and extensive infusion of such lipid emulsions may thus increase the plasma concentration of free AA by one order of magnitude, leading to an alteration of the eicosanoid profile, deterioration of the oxygenation index and ventilation-perfusion mismatching in the lung. In contrast to Intralipid, Clinoleic is rich in monounsaturated fatty acids (MUFAs), mostly in the form of oleic acid found in olive oil, lower amounts of PUFAs, and long-chain only triglycerides [15]. Omegaven is rich in n-3 PUFAs, in particular eicosapentaenoic acid (EPA) and docosahexaenoic acid (DHA), and has a very low concentration of LA. The administration of n-3 PUFAs, such as FO, may represent a novel promising strategy for enriching nutrition regimens, as it has been demonstrated to modulate excessive inflammatory reactions in experimental animals, healthy volunteers and subjects in clinical trials [16].

Cytokines play a key role in the promotion or inhibition of ALI, but the mechanism underlying cytokine regulation is complex. In this study, we sought to investigate the possible benefits of using a different lipid emulsion in rats against LPS-induced ALI, focusing on cytokine production and other related events. Our results demonstrated that FO-based lipid emulsion preconditioning of rats attenuates the production of several vital pro-inflammatory factors in the LPS-induced ALI model, and improves liver function.

## Materials and methods

### Materials

Clinoleic rich in MUFAs was obtained from Baxter/Clintec Parenteral SA (Cedex, France), Intralipid rich in ω-6 PUFAs and Omegaven rich in ω-3 PUFAs were purchased from Sino-Swed Pharmaceutical Corp. Ltd. (Jiangsu, China), and LPS (*Escherichia coli* 0111:B4) was provided by Sigma-Aldrich; Merck KGaA (St. Louis, MO, USA).

### Animals

The experimental protocol was approved by the Animal Care and Use Committee of Wenzhou Medical University (Zhejiang, China). Male Sprague-Dawley rats weighing 160–200 g were purchased from Slyke’s Animal Center [Shanghai, China; license no. SCXK (Hu) 2003–0003] and allowed to acclimatize under specific pathogen-free conditions for 1 week prior to the beginning of the experiment. Animal care, including ad libitum access to chow and water, conformed to the guidelines of the National Institutes of Health and the Chinese National guidelines on animal experimentation.

### ALI model

The rats were preconditioned for 7 days with saline or lipid emulsion by injecting 30 ml/kg per day via the tail vein of 20% Intralipid (6 g/kg body weight per day), 20% Clinoleic (6 g/kg), 10% Omegaven (3 g/kg), or 0.9% NaCl, respectively; then randomly divided into 5 groups (*n* = 15 per group) as follows: Intralipid group (preconditioned with Intralipid/challenged with LPS), Clinoleic group (preconditioned with Clinoleic/challenged with LPS), Omegaven group (preconditioned with Omegaven/challenged with LPS), LPS group (preconditioned with saline/challenged with LPS) and saline group (both preconditioned and challenged with saline). The above emulsion doses were derived according to the manufacturer’s instruction, doubled than clinical usage. As previously described [17], ALI was induced on the 8th day by intratracheal administration of LPS (0.5 mg/kg, 2 g/l, 0.04–0.05 ml) which was slowly injected into the trachea using a 4.5-mm needle. Control rats were intratracheally administered 0.25 ml sterile normal saline. ALI was blindly scored according to previously described criteria [18], and each tissue sample was graded on a four-point scale (0 to 3), with higher scores (averaged from 10 high-power fields) indicating more severe damage.

### Sample collection

Samples were collected 8 h after LPS treatment. Blood samples (4–5 ml) without anticoagulant were maintained at room temperature (RT) for 2 h and centrifuged later at 2000 rpm for 15 min at RT to separate the serum, which was used to measure prostaglandin E_2_ (PGE_2_), leukotriene B_4_ (LTB_4_) and liver enzymes. Bronchoalveolar lavage fluid (BALF) was collected as described [17], and every 100 μl of BALF was added 1 *μ*l of proteinase inhibitor cocktail (Sigma-Aldrich; Merck KGaA), kept on ice, centrifuged for 15 min at 2000 rpm, and each supernatant was collected and stored at − 80 °C prior to the cytokine assays. Portions of the lung lobes (anterior, median and posterior lobe of the right lung; upper and lower lobe of the left lung) were harvested and divided equally for the histological examinations and the biochemical measurements. For TUNEL analysis, unlavaged lungs were fixed in 4% paraformaldehyde overnight, embedded in paraffin, and cut into 4-μm tissue sections. Central sections from the right lung were cut, collected in cryotubes, flash-frozen by immersion in liquid nitrogen, and stored at − 80 °C.

### Polymerase chain reaction (PCR) analysis

Total RNA extracted from frozen tissues using TRIzol reagent (Invitrogen; Thermo Fisher Scientific Inc., Shanghai, China) was measured by spectrophotometry in a Nanodrop® ND-1000 system (Thermo Fisher Scientific). The primers for PCR were purchased from Invitrogen; Thermo Fisher Scientific Inc. First-strand cDNA was synthesized at 42 °C for 1 h, 95 °C for 5 min and 4 °C for 10 min from 0.5 μg/sample total RNA using an M-MLV Reverse Transcriptase Kit (TaKaRa Biomedical Technology Co. Ltd., Beijing, China). The following primers for IL-1β (sense 5′-GCAACTGTCCCTGAACTCAACT-3′ and antisense 5′-TTGTCGAGATGCTGCTGTGA-3′), IL-6 (sense 5′-AACGATGATGCACTTGCAGA-3′ and antisense 5′-GGAAATTGGGGTAGGAAGGA-3′) and GAPDH (sense 5′-GGT GAA GGT CGG TGTG AAC-3′ and antisense 5′-CGT TGA TGG CAA CAA TGT C-3′) were purchased from JiKang Biotech Company (Shanghai, China). The PCR conditions were 94 °C for 5 min, 94 °C for 30 s, and 72 °C for 5 min. Different cycles were carried out according to different primers. Finally, 5–8 μl reaction products were electrophoresed on 1% agarose gel, and then stained with ethidium bromide to observe the amplified DNA bands under an ultraviolet lamp.

### Enzyme-linked immunosorbent assay (ELISA)

The levels of IL-1β, IL-6, TNF-α, PGE_2_ and LTB_4_ were measured using commercially available ELISA kits (all from Shanghai Westang Biotech Co., Ltd.) according to the manufacturers’ instructions. Optical density was measured at 450 nm using an ELISA microplate reader.

### Terminal deoxynucleotidyl transferase dUTP nick end labeling (TUNEL) staining

Paraffin-embedded tissue slices were dewaxed, washed with PBS and digested with proteinase K in a wet box for 15 min at 37 °C. After a repeat PBS wash, the slides were dipped in TUNEL reaction mixture (Roche Diagnostics GmbH, Mannheim, Germany) and then incubated for 1 h at 37 °C in the wet box. After washing, the sections were incubated with converter-AP for 30 min at 3 °C in the wet box, and then washed with PBS. Subsequently, the sections were stained with 3,3 diaminobenzidine, re-dyed with hematoxylin, and the signals were observed under a microscope. All cells with brown nuclei were considered to be apoptotic. Apoptotic cells were counted at a magnification of × 400 in 5 random, non-overlapping fields per section. Apoptosis index was calculated as number of apoptotic cells/number of total cells × 100%.

### Liver enzymes

Alanine aminotransferase (ALT) and aspartate aminotransferase (AST) were measured using commercial kits, with an automatic biochemical analyzer (OLYMPUS AU7100; Olympus Corporation, Tokyo, Japan) provided by the Laboratory of The First Affiliated Hospital of Wenzhou Medical University.

### Statistical analyses

Statistical analyses were performed with Sigmaplot software (SPSS version 20.0; IBM Corp., Armonk, NY, USA). Each point corresponds to mean ± standard deviation. Comparisons between two groups were made with the two-tailed Fisher’s exact *t*-test. Comparisons between multiple groups were made by one-way analysis of variance. For post hoc analysis, the Fisher’s test was used. A value of *P* < 0.05 was considered to indicate statistically significant differences.

## Results

### Animal status during lipid emulsion conditioning and ALI induction by LPS challenge

Rats were generally in good condition during lipid emulsion conditioning and LPS challenge. A total of 5 groups (*n* = 15 per group) were finally created.

### Histopathology of lung injury of the ALI rats preconditioned with different lipid emulsions

**Fig. 1:**
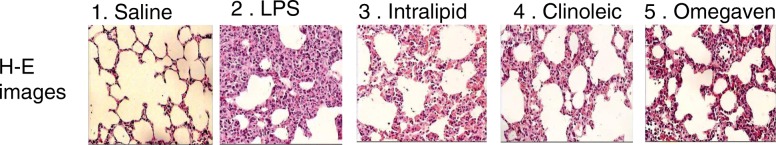
Representative images of H&E staining in the lung (Magnification × 200) of the ALI rats preconditioned with different lipid emulsions. There were intra-alveolar/interstitial patchy hemorrhages, interstitial edema, and infiltration of inflammatory cells around the bronchus in all the rats challenged with LPS. A1, Saline; A2, LPS; A3, Intralipid; A4, Clinoleic; A5, Omegaven

Compared to the almost normal histology of the saline group at 8 h post-challenge with saline, all the rats challenged with LPS exhibited intra-alveolar/interstitial patchy hemorrhages, interstitial edema and peribronchial infiltration of inflammatory cells, without any significant difference among the groups (Fig. 1). The mean ALI score of the rats without lipid supplementation was (9.2 ± 1.5), and the mean ALI score of the rats given parental nutrition (PN) with soybean oil was (7.3 ± 0.7). By contrast, the ALI score of the rats given PN supplemented with FO was (6.6 ± 0.6), and the ALI score of the rats given PN supplemented with olive oil was (6.3 ± 0.7). The scores of the four LPS-challenged groups were significantly higher compared with that of the saline group, suggesting that the LPS-induced ALI model was successfully established.

### IL-1 β, IL-6 and TNF-α in lung and/or BALF of the ALI rats preconditioned with different lipid emulsions upon LPS challenge

**Fig. 2:**
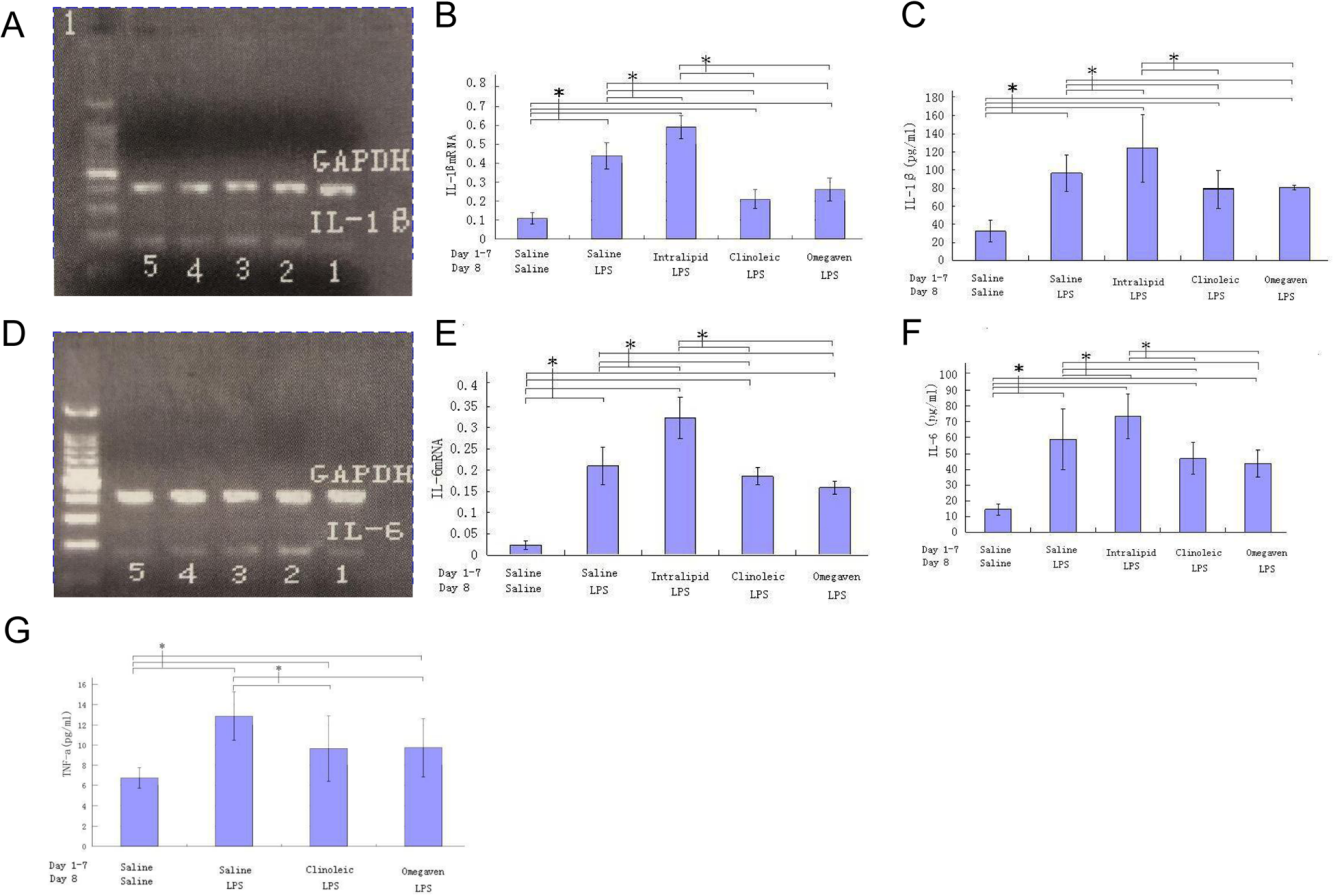
Measurement of IL-1 β and IL-6 in lung tissue and bronchoalveolar lavage fluid (BALF) of the ALI rats preconditioned with different lipid emulsions. Rats were preconditioned with different lipid emulsions for one week, and BALF supernatants and lung tissues were collected 8 h after ALI induction from LPS challenge. **a**, **b**, qRT-PCR test of IL-1β in lung tissue, band intensity (means for lanes 1–5, 0.111 ± 0.023; 0.435 ± 0.070; 0.592 ± 0.060; 0.214 ± 0.046; and 0.256 ± 0.060 for groups Saline, LPS, Intralipid, Clinoleic, Omegaven, respectively) was quantified with software; **c**, ELISA test of IL-1β in BALF. **d**, **e**, qRT-PCR test of IL-6 in lung tissue, band intensity (means for lanes 1–5, 0.023 ± 0.010; 0.214 ± 0.043; 0.323 ± 0.056; 0.183 ± 0.020; 0.151 ± 0.015 for groups Saline, LPS, Intralipid, Clinoleic, Omegaven, respectively) was quantified with software; **f**, ELISA test of IL-6 in BALF. **g**, Measurement of TNF-α with ELISA in bronchoalveolar lavage fluid (BALF). Results were expressed as the means ± SD from the PCR band quantification or the corresponding protein level relative to ELISA reading (number of rats per group, Saline 16, LPS 17, Intralipid 15, Clinoleic 16, and Omegaven 16). *P < 0.05 (ANOVA)

IL-1β gene expression was measured by PCR and quantification of band intensity revealed that the IL-1β mRNA level in the lung tissue from the LPS group was higher (*P* < 0.05) compared with those in the saline, Clinoleic or Omegaven groups, but lower compared with that in the Intralipid group (Fig. 2a and b). Similarly, the IL-1β protein level in the BALF sample measured by ELISA was higher (P < 0.05) in the LPS group compared with those in the saline, Clinoleic or Omegaven groups, but lower compared with that in the Intralipid group (Fig. 2c).

With different values but similar trend, the IL-6 mRNA expression in the lung (Fig. 2d and e) and the IL-6 protein level in the BALF (Fig. 2f) were highest in Intralipid group rats, followed by the LPS, Clinoleic and Omegaven groups.

The TNF-α level in the BALF sample of the LPS group was significantly higher compared with that of the Clinoleic or Omegaven groups, although unfortunately we were unable to make a comparison to the Intralipid group due to small sample (Fig. 2g).

### Apoptosis of epithelial lung cells of the ALI rats preconditioned with different lipid emulsions upon LPS challenge

**Fig. 3:**
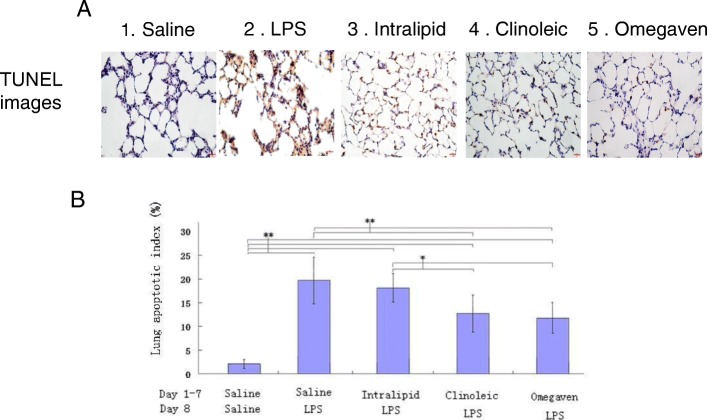
Apoptotic index (AI) in lung tissue samples of the ALI rats preconditioned with different lipid emulsions. Lung tissue sections were stained with TUNEL reagents (**a**1, Saline; **a**2, LPS; **a**3, Intralipid; **a**4, Clinoleic; **a**5 Omegaven) and positive (brown) cells in 500 epithelial cells from 5 randomly-selected areas per section under a microscope (Magnification × 400) were counted by pathologists unaware of experiment grouping and the numbers were used to calculate the apoptotic index (**b**). Results in B were expressed as the means ± SD (number of rats per group, Saline 16, LPS 17, Intralipid 15, Clinoleic 16, and Omegaven 16).*P < 0.05, **P < 0.01 (ANOVA)

The apoptosis was measured by TUNEL assay and the results demonstrated that lung tissue sections of rats from the saline + LPS treatment group included significantly more apoptotic cells compared with the Clinoleic or Omegaven groups (Fig. 3).

### Serum level of PGE_2_ and LTB_4_ of the ALI rats preconditioned with different lipid emulsions upon LPS challenge

**Fig. 4:**
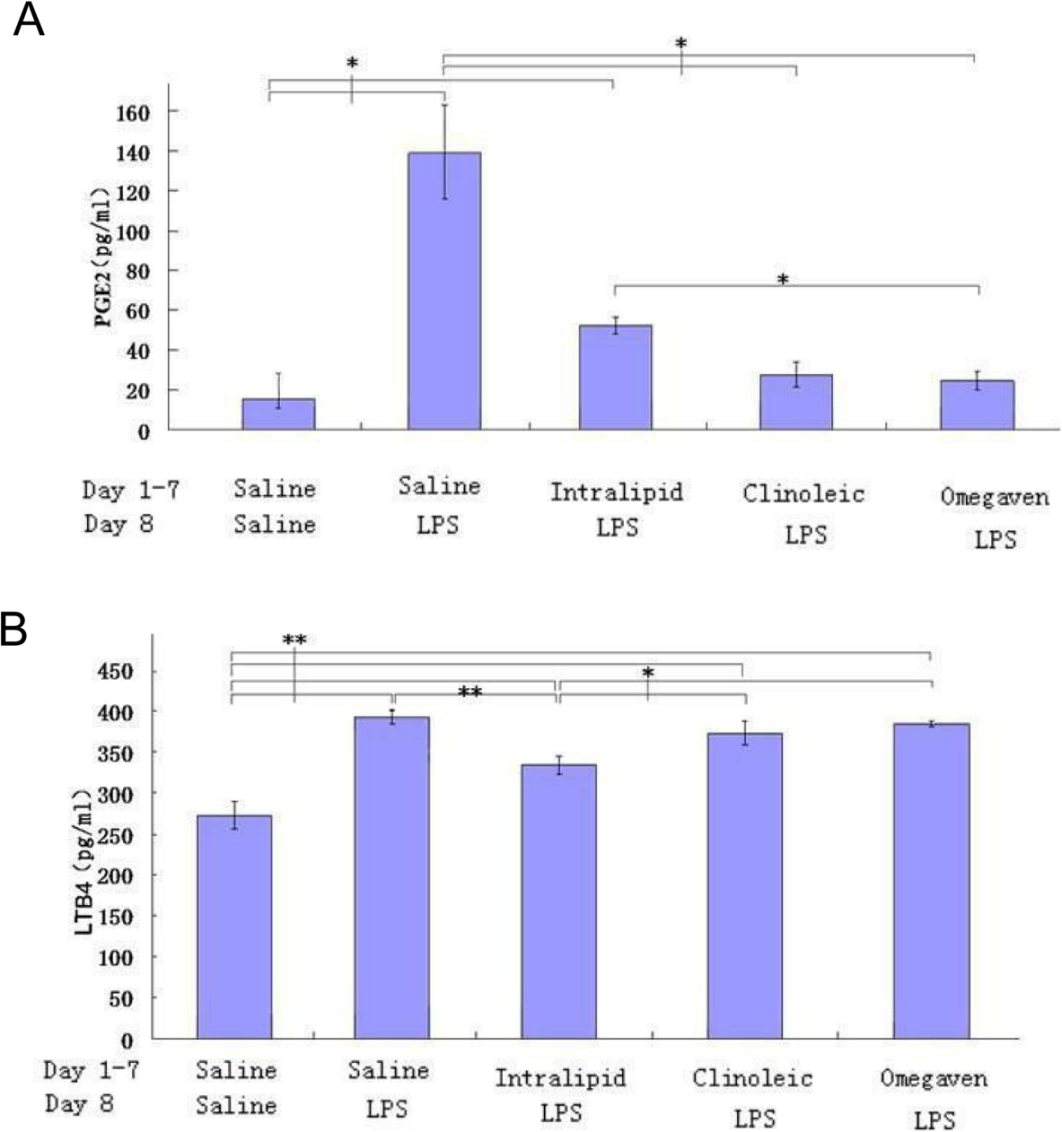
Representative biomarkers showing Omegaven preconditioning reduces the inflammation of ALI rats. Prostaglandin E_2_ (PGE_2_) and leukotriene B_4_ (LBT_4_) in serum was measured with ELISA. **a**, PGE_2_ value; **b**, LBT_4_ value. Results were expressed as the means ± SD (number of rats per group, Saline 16, LPS 17, Intralipid 15, Clinoleic 16, and Omegaven 16).*P < 0.05 (ANOVA)

PGE_2_ production in the serum was highest in the LPS group, followed by the Intralipid group, and the PGE_2_ level of these two groups was significantly (*P* < 0.05) higher compared with those of the other groups (Fig. 4a). Interestingly, Intralipid conditioning was associated with a significantly lower production of LTB_4_ compared with the LPS, Clinoleic, or Omegaven groups (Fig. 4b).

### Liver function evaluation of the ALI rats preconditioned with different lipid emulsions

**Fig. 5:**
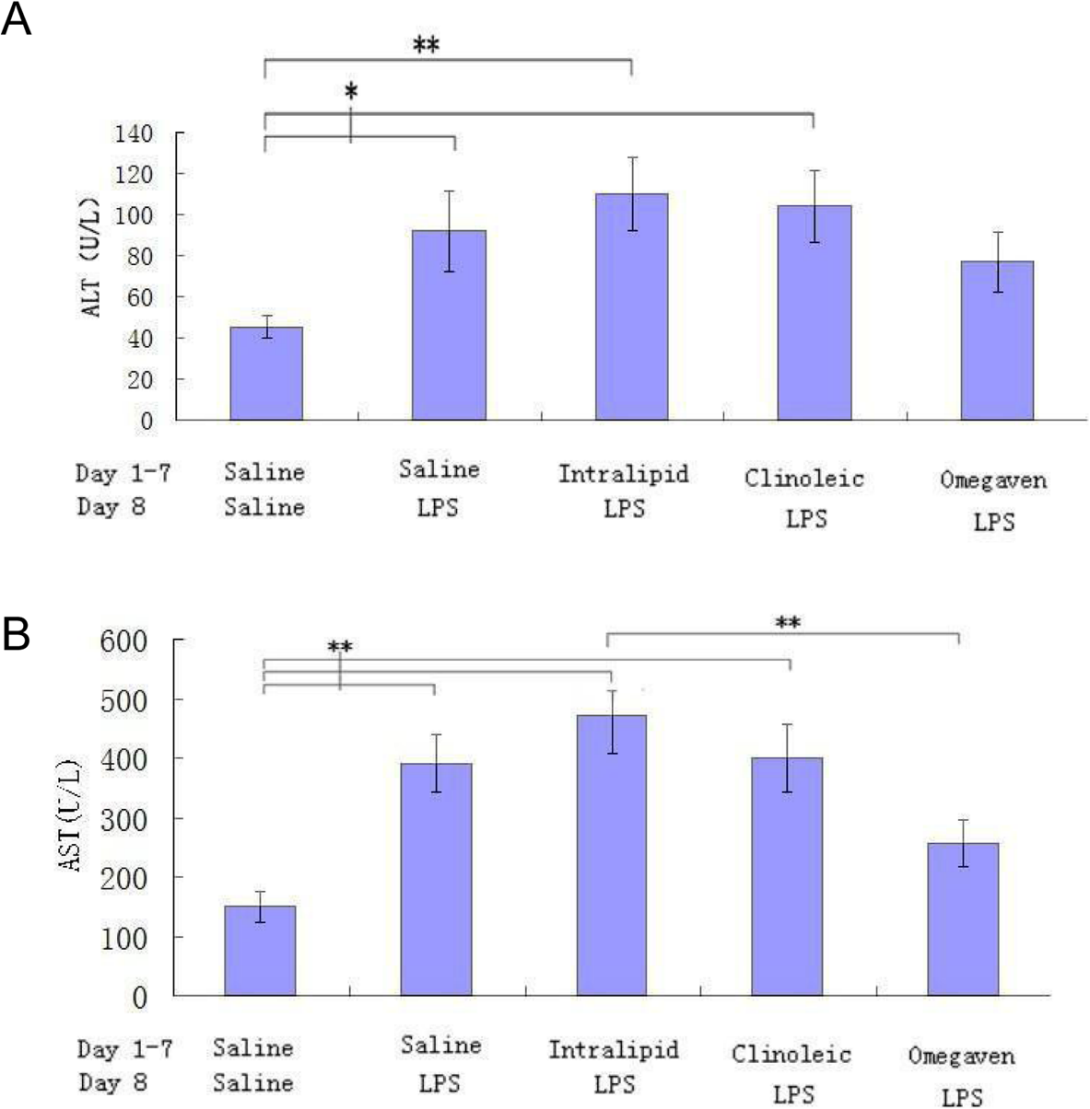
Representative enzyme markers showing Omegaven preconditioning attenuates liver malfunction of the ALI rats preconditioned with different lipid emulsions. Aspartate aminotransferase (AST) and alanine aminotransferase (ALT) in serum were measured with OLYMPUS AU7100 automatic biochemical analyzer. **a**, ALT value; **b**, AST value. Results were expressed as the means ± SD (number of rats per group, Saline 16, LPS 17, Intralipid 15, Clinoleic 16, and Omegaven 16). *P < 0.05, **P < 0.01 (ANOVA)

Increased plasma levels of ALT and AST are established indicators of liver damage. Omegaven preconditioning slightly increased ALT, but the difference from the saline group was not significant; however, the ALT values in the other three LPS-challenged groups were significantly higher compared with the saline group (Fig. 5a). Plasma AST exhibited similar changes (Fig. 5b), and Omegaven preconditioning was associated with a significantly less prominent AST increase (*P* < 0.01) compared with Intralipid preconditioning.

## Discussion

In the present study, we investigated the impact of three different commercially available lipid emulsions in a rat model of ALI. The results demonstrated that supplementing PN with FO significantly reduced LPS-induced ALI in rats, as determined by reduction of lung inflammation and protective effect on liver function.

Prostaglandins and leukotrienes are known potent inflammatory lipid mediators that are involved in hypersensitivity and respiratory disorders, and have immunoregulatory properties [19, 20]. AA is metabolized to produce proinflammatory series 4 leukotrienes and series 2 prostaglandins. EPA and DHA are precursors of series 5 leukotrienes and series 3 prostaglandins, 4 resolvins, defensins and maresins, which are anti-inflammatory mediators [21–23]. Although conditions may vary, IL-1β, IL-6 and TNF-α are often considered as the typical early-phase cytokines implicated in the pathogenesis of ALI [24, 25].

Previous research investigated the kinetics of cytokine production in similar ALI models. In an ALI rat model caused by cecal ligation and puncture-induced sepsis, two independent laboratories demonstrated that the protein levels of TNF-α and IL-6 in BALF measured by ELISA were highest at 6 h and declined at later time points (12 and 24 h) [26, 27]. In a mouse model of ALI induced by intraperitoneal LPS injection, the mRNA expression of TNF-α in lung tissues peaked at 6 h and decreased thereafter [28]. In the present study, at 8 h post-LPS challenge, rats preconditioned with Intralipid exhibited the highest levels of proinflammatory factors in lung tissues and the highest plasma AST level among all groups, and were 2nd only to the LPS group regarding lung cell apoptosis and plasma PGE_2_ level. Although the exact levels of these parameters soon after the end of preconditioning (day 7) are not known, Jing et al [29] reported that the levels of TNF-α, IL-1β, IL-6 in the lung tissue remained the same before and after perfusion with FO when compared between FO preconditioned and unconditioned rats; the same may be assumed to be the case at 7 days post-preconditioning, at least for the Omegaven group. It is reasonable to hypothesize that Intralipid preconditioning for 1 week primed the rats to respond to LPS challenge the next day to produce even more proinflammatory factors compared with the LPS group.

Despite their distinct difference in composition, preconditioning with Clinoleic or Omegaven exerted similar protective effects in rats against LPS challenge, with significantly lower IL-1β and IL-6 levels and lower apoptosis rate compared with the Intralipid and the LPS groups. Supporting our findings, FO emulsion preconditioning for 3 days was previously found to significantly lower the serum and lung levels of TNF-α, IL-1β and IL-6 in an ischemia-reperfusion ALI model in rats [29]. Clinoleic or Omegaven preconditioning also reduced TNF-α in BALF in the present study, similar to previous findings from humans [7], rats [19, 29] and mice [30, 31], indicating that infusion of FO achieved a significant reduction of TNF-α upon ALI induction.

Although the exact mechanism of apoptosis in ALI remains to be investigated in more detail, several highly relevant studies [32–34] reported that increased alveolar epithelial cell apoptosis plays a central role in the development and progression of ALI. Although a different mechanism (ischemia-reperfusion) was used to induce ALI in male Wistar rats, pretreatment with ω-3 PUFAs effectively inhibited apoptosis of the lung epithelium as reflected in the changes of p66shc phosphorylation, a pro-apoptosis marker [29], which was consistent with our finding that preconditioning with ω-3-rich Omegaven significantly lowered the lung apoptosis index upon LPS challenge.

Surprisingly, Intralipid conditioning was associated with the lowest LTB_4_ level among the four LPS-challenged groups, with a significant (*P* < 0.05) difference (Fig. 4b), suggesting that Intralipid may modulate the synthesis or metabolism of LTB_4_, and its role in ALI would be balanced by other factors, or it may reflect a partitioning of AA towards the cyclo-oxygenase (COX)-2 rather than the 5-lipoxygenase pathway, which may be due to greater induction of COX-2 compared with lipoxygenase in this model, which warrants further investigation.

Although ALI per se may cause significant liver injury [35], one of the risks of using lipid emulsion as parenteral nutrition is also liver disease [36]. Therefore, the lipid emulsion must be selected with caution if an ALI patient requires parenteral nutrition, and liver function should be continuously monitored. Our finding that preconditioning with Omegaven was associated with the lowest ALT/AST level among the four LPS-challenged groups appears to be important in this regard, and requires further detailed investigation in terms of kinetics and underlying molecular mechanisms. Based completely on FO without any phytosterols, Omegaven is rich in ω-3 and has hepatoprotective properties [14], in contrast to Intralipid that has a higher ω-6 polyunsaturated fatty acid and phytosterol content [14], which may be hepatotoxic; these may be the reasons why these two emulsions had different performance regarding liver function in the present study.

There were a few limitations in our study. To minimize the possibility of causing local tissue necrosis, a low end of LPS dose was chosen. To minimize the number of animals, only one dose of lipid emulsion for preconditioning, LPS for challenge, a single timepoint for ending the experiment, all based on literature research. The emulsion doses were comparable to those (1.5 g/kg/d) used in reference [31]. Since no mechanical ventilation machine was available for experimental animals, a reasonable timepoint (8 h after LPS treatment) was chosen to ensure the survival of the challenged rats. Events before or after that point, although speculated, were discussed above.

## Conclusions

To summarize, in one of the typical rat ALI settings, preconditioning with Omegaven or Clinoleic was superior to Intralipid in decreasing the intensity of the cytokine storm and apoptosis caused by LPS challenge; in addition, Omegaven had the potential to improve liver function. The results from the present study set a basis for further investigation of the molecular mechanisms of ALI, including the up- and downstream pathways of proinflammatory factor production, in search of (small) molecules intervening with the pathogenesis of ALI in order to translate relevant research findings into clinical benefit for patients with ALI. The use of Omegaven or Clinoleic, particularly in patients with ALI, is still characterized by uncertainty due to a lack of relevant studies. Future investigations must specifically focus on the route of administration and mode of application (enteral vs. parenteral/bolus vs. continuous), determining an optimal dose of Omegaven or Clinoleic, and the defining the best timepoint(s) for administration.

## Data Availability

All data and material can be provided upon request.

## Abbreviations

AA: Arachidonic acid
ALI: Acute lung injury
ALT: Alanine aminotransferase
AST: Aspartate aminotransferase
DHA: Docosahexaenoic acid
ELISA: Enzyme-linked immunosorbent assay
EPA: Eicosapentaenoic acid
LPS: Lipopolysaccharide
LTB_4_: Leukotriene B_4_
MUFAs: Monounsaturated fatty acids
PCR: Polymerase chain reaction
PGE_2_: Prostaglandin E_2_
PN: Parental nutrition
PUFA: Polyunsaturated fatty acid
TUNEL: Terminal deoxynucleotidyl transferase dUTP nick end labeling
